# Titanium Wear Particles Exacerbate *S. epidermidis*-Induced Implant-Related Osteolysis and Decrease Efficacy of Antibiotic Therapy

**DOI:** 10.3390/microorganisms9091945

**Published:** 2021-09-13

**Authors:** Claudia Siverino, Linda Freitag, Daniel Arens, Ursula Styger, R. Geoff Richards, T. Fintan Moriarty, Vincent A. Stadelmann, Keith Thompson

**Affiliations:** 1 AO Research Institute Davos, 7270 Davos-Platz, Switzerland; claudia.siverino@aofoundation.org (C.S.); linda-freitag@gmx.de (L.F.); daniel.arens@aofoundation.org (D.A.); ursula.styger@aofoundation.org (U.S.); geoff.richards@aofoundation.org (R.G.R.); fintan.moriarty@aofoundation.org (T.F.M.); vincent.stadelmann@kws.ch (V.A.S.); 2 Department of Teaching, Research and Development, Schulthess Clinic, 8008 Zürich, Switzerland

**Keywords:** *Staphylococcus epidermidis*, osteomyelitis, microCT, wear particles, antibiotics

## Abstract

Total joint arthroplasty (TJA) surgeries are common orthopedic procedures, but bacterial infection remains a concern. The aim of this study was to assess interactions between wear particles (WPs) and immune cells in vitro and to investigate if WPs affect the severity, or response to antibiotic therapy, of a *Staphylococcus epidermidis* orthopedic device-related infection (ODRI) in a rodent model. Biofilms grown on WPs were challenged with rifampin and cefazolin (100 µg/mL) to determine antibiotic efficacy. Neutrophils or peripheral blood mononuclear cells (PBMCs) were incubated with or without *S. epidermidis* and WPs, and myeloperoxidase (MPO) and cytokine release were analyzed, respectively. In the ODRI rodent model, rats (*n* = 36) had a sterile or *S. epidermidis*-inoculated screw implanted in the presence or absence of WPs, and a subgroup was treated with antibiotics. Bone changes were monitored using microCT scanning. The presence of WPs decreased antibiotic efficacy against biofilm-resident bacteria and promoted MPO and pro-inflammatory cytokine production in vitro. WPs exacerbated osteolytic responses to *S. epidermidis* infection and markedly reduced antibiotic efficacy in vivo. Overall, this work shows that the presence of titanium WPs reduces antibiotic efficacy in vitro and in vivo, induces proinflammatory cytokine release, and exacerbates *S. epidermidis*-induced osteolysis.

## 1. Introduction

Total joint arthroplasty (TJA) is one of the most successful surgical procedures offering improved quality of life for patients with pain or functional limitations due to conditions such as osteoarthritis [1]. There are more than 1 million TJAs performed in the United States annually, and this number is expected to increase to nearly 4 million by 2030 [2]. However, complication rates following primary hip replacement, for example, range between 2 and 10% [3]. While a large proportion of these complications are due to either aseptic loosening (36.5%) and prosthesis dislocation (17.7%), up to 15% are due to periprosthetic joint infections (PJIs), with the remaining complications being connected to other etiologies [4]. Treatment of PJI often involves revision surgery, which has an even higher postoperative complication rate and increased duration of hospital stays [5]. The incidence of PJIs in primary total hip arthroplasties is 0.2–2%, while for revision surgeries it may reach 5% [6]. PJI is a potentially devastating complication that can result in implant failure, prolonged and challenging treatment involving further loss of bone and, in extreme cases, may require amputation of the affected limb [7,8].

Most PJI cases are caused by perioperative inoculation of micro-organisms [9]. Early infections manifest within the first four weeks after implantation, while delayed infections may appear between three months and three years. Highly virulent pathogens (e.g., *Staphylococcus aureus*, streptococci, enterococci) typically result in acute infections presenting with evident local and systemic evidence of inflammation. Conversely, delayed infections are typically caused by low-virulence organisms, such as coagulase-negative *Staphylococcus* spp. (CoNS) or *Cutibacterium* species [9,10]. *S. epidermidis*, the most common CoNS species recovered from clinical cultures, colonizes human epithelial surfaces [11]. While usually innocuous or even beneficial colonizers, once the host epithelial barrier is compromised, CoNS such as *S. epidermidis* can cause serious infections. In fact, CoNS infections account for the majority of bacterial sepsis associated with inserted or implanted medical devices, with *S. epidermidis* being the most significant species in that regard [12].

Upon encountering an implant surface, bacteria rapidly form a biofilm, which is a complex 3D community consisting of clusters of one or several species of micro-organisms living together in a highly hydrated, self-produced extracellular matrix [13]. Bacteria resident in such biofilms enter a metabolically dormant state, rendering them markedly less sensitive to conventional antibiotic treatment [13]. In addition, the extracellular matrix of the biofilm also prevents immune cell invasion, which, together with the decreased phagocytic activity of immune cells induced by the presence of the implant itself, presents a challenging environment for the effective treatment of PJIs in patients [14].

The long-term survival rates of joint replacement implants are also affected by their wear resistance and the biologic reactivity of the generated wear particles (WPs) [15]. Despite the biocompatibility of the material used to fabricate joint replacement implants, such as metals, polymers and ceramics, the WPs generated at the implant surface can trigger biological responses [16,17]. Factors such as WP size, morphology, and material composition influence their biologic reactivity and are important determinants of peri-implant cell fate [18]. In particular, polymeric and metal debris represent the major concerns, while ceramic debris displays a relatively low incidence of osteolysis [17,19,20]. The inflammatory response caused by the WPs involves several cell types and cytokines, which then induce periprosthetic osteolysis and consequent aseptic implant loosening [21]. Macrophages represent the main cell type present at the interface of tissue surrounding the loosened prosthesis, but other cells such as fibroblasts, osteoblasts, osteoclasts, lymphocytes and polymorphonuclear cells such as neutrophils are also observed [22]. Following exposure to WPs, macrophages express several chemokines and pro-inflammatory cytokines, such as interleukins (IL-1 and IL-6) and tumor necrosis factor alpha (TNF-α), which lead to further recruitment of macrophages and induces osteoclast formation from monocyte/macrophage lineage precursors, thereby increasing bone resorption [23].

The influence of WPs in PJI and whether WPs exacerbate infection and consequent inflammatory responses and osteolysis in vivo have not been widely investigated. Given the additional surface area of WPs for providing a further substrate for bacterial adhesion and growth [24], we sought to examine how WPs affect the growth and antibiotic susceptibility of *S. epidermidis* in vitro and assess the immunomodulatory effects of WPs on immune cells. In addition, we also determined the impact of WPs on the severity of a *S. epidermidis* orthopedic device-related infection (ODRI) and its response to antibiotic therapy in our established longitudinal in vivo microCT-based rat model [25,26]. Our findings show that the presence of WPs diminishes antibiotic efficacy in vitro and promotes pro-inflammatory cytokine production by immune cells. Furthermore, titanium WPs promote *S. epidermidis*-induced osteolysis and reduce the efficacy of antibiotic therapy in vivo.

## 2. Materials and Methods

### 2.1. Materials

Titanium powder (<20 µm diameter; #00681) was purchased from Alfa Aesar (Karlsruhe, Germany) and was used as the source of WPs throughout the study. WPs were heat sterilized at 230 °C for 1 h to remove endotoxin contamination, prior to reconstitution in sterile PBS and sonication for 3 min using a Bandelin Ultrasonic water bath (Model RK 510 H) prior to use. The efficacy of endotoxin removal from the WPs was confirmed using the ToxinSensor^TM^ EndoToxin Detection System (GenScript, Leiden, The Netherlands). All other reagents/chemicals were purchased from Sigma-Aldrich (Buchs, Switzerland) unless otherwise stated.

### 2.2. Antibiotic Challenge of S. epidermidis Grown on Titanium WPs

*S. epidermidis* (strain 103.1; a clinical isolate from a patient with a chronic ODRI and available from the Culture Collection of Switzerland, strain number CCOS 1152) was recovered from frozen stocks (−80 °C in 20% (*v*/*v*) glycerol) and cultured in tryptic soy broth (TSB, Oxoid, Basel, Switzerland) overnight in ambient air at 37 °C with agitation at 100 rpm. The sensitivity of this strain (minimum inhibitory concentration (MIC) values) to rifampicin and cefazolin was 0.015 and <1 µg/mL, respectively. The following day, the overnight culture was diluted to 1:20 in TSB and cultured as described above for 2 h. The *S. epidermidis* culture was then adjusted to an optical density of 0.001 at 600 nm (corresponding to ~5 × 10^5^ CFU/mL) using a spectrophotometer (Multiskan, Thermo Fisher Scientific, Switzerland). A total of 5 × 10^4^ CFUs of *S. epidermidis* (100 µL) were then cultured alone, or in the presence of 0.1 or 1 mg/mL of WPs in 96-well plates (TPP Techno Plastic Products AG, Switzerland). Bacteria were cultured for 24 h in ambient air at 37 °C on a rocking platform to generate a biofilm. The supernatant was then carefully removed and fresh TSB-containing antibiotics (a combination of 100 µg/mL rifampin and 100 µg/mL cefazolin) was added for a further 24 h. The plate was then sonicated for 10 min to remove adherent bacteria, then the bacteria including WPs were immediately collected, washed in PBS, and centrifuged at 9000× *g* for 5 min. After resuspension in PBS, serial dilutions were performed on tryptic soy agar (TSA) plates to quantify the surviving bacterial population.

### 2.3. Confirmation of Biofilm Formation by Scanning Electron Microscopy

Cultures of *S. epidermidis* grown on 1 mg/mL WPs in the absence or presence of antibiotic treatment (100 µg/mL rifampin and 100 µg/mL cefazolin) for 24 h were prepared on glass coverslips for subsequent SEM analysis. Prior to imaging, cultures were washed with PBS to remove non-adherent bacteria, leaving only biofilm-resident structures. The samples were fixed using 70% ethanol for 2 days and then dehydrated in an ascending ethanol series (80, 90, 96 and 100%: 10 min per solution). The ethanol was then removed, and samples were air dried at room temperature. The samples were then sputter coated with 10 nm gold and palladium using a BAL-TEC MED 020 sputter coater connected to a BAL-TEC MCS 010 Multi Control System and a BAL-TEC QSG 060 Quartz Thickness and Rate Monitor (both BAL-TEC AG, Liechtenstein). SEM images of the surface were acquired using a Hitachi S-4700 FESEM (accelerating voltage = 3 kV; 40 µA current; working distance = 12 mm) and a Quartz PCI image management system.

### 2.4. Assessment of Antibacterial Efficacy of Neutrophils

Blood was obtained from healthy donors following informed consent according to the Declaration of Helsinki and local approval (Ethics Commission Zürich: Approval Number 2019-02353). Neutrophils were isolated using an EasySep™ Direct Human Neutrophil Isolation Kit, following the manufacturer’s instructions. The negatively selected neutrophils were diluted in RPMI 1640 medium (Gibco) supplemented with 10% fetal bovine serum (FBS; Corning), subsequently referred to as complete RPMI, to a final concentration of 6 × 10^6^ cells/mL. A *S. epidermidis* overnight culture was diluted to 1:20 in TSB and cultured (ambient air at 37 °C with agitation at 100 rpm) for 2 h. The logarithmically growing *S. epidermidis* culture was then centrifuged (9000× *g* for 5 min) and resuspended in complete RPMI medium, and the optical density at 600 nm was measured and adjusted to 0.1 using a spectrophotometer (Multiskan). From OD_600_ = 0.1, serial 1:10 dilutions were performed to reach 0.00001 (~10^4^ CFU/mL, experimentally determined). Finally, 10^2^ CFUs of *S. epidermidis* were incubated with 6 × 10^5^ neutrophils, in the presence or absence of 0.1 or 1 mg/mL of WPs, and incubated at 37 °C, 5% CO_2_ for 3, 6 and 24 h. At each time point, plates were placed on ice to inhibit neutrophil killing activities. Suspensions of neutrophils plus bacteria were harvested, centrifuged for 5 min at 250× *g* at 4 °C, and the supernatant was collected to recover extracellular bacteria. A further wash in PBS was then performed to recover additional extracellular bacteria. The cell pellet containing the neutrophils was then incubated at room temperature for 10 min with a neutrophil lysing solution (40 μL of 4% (*v*/*v*) Tween 20 in ddH_2_O) followed by the addition of 60 μL of ddH_2_O [27] to recover intracellular bacteria. The collected supernatants with either extracellular or intracellular bacteria were diluted to spot inoculate 10 µL of each dilution on TSA plates to quantify the surviving population.

### 2.5. WP-Induced Soluble Mediator Production by Neutrophils and PBMCs

Myeloperoxidase (MPO) release from neutrophils (5 independent donors), cultured in the absence or presence of WPs, was assessed after 24 h using a U-PLEX MPO assay and a MESO QuickPlex SQ120 plate-reader (both Meso Scale Diagnostics, Rockville, MD, USA). To trigger neutrophil activation, parallel cultures in the presence of the chemotactic peptide N-formyl-Mel-Leu-Phe (FMLP) were also performed.

Peripheral blood mononuclear cells (PBMCs) (3 independent donors) were cultured overnight at a density of 1 × 10^6^ cells/mL in complete RPMI 1640 medium supplemented with 100 U/mL penicillin and 100 µg/mL streptomycin (Gibco). The following day, 0.1 or 1 mg/mL WPs and/or 10 µg/mL lipoteichoic acid (LTA; as a stimulus representative of Gram-positive bacteria) were added. After 24 h, PBMCs were harvested (400× *g* for 5 min), and the conditioned medium was collected and assessed for a range of pro- and anti-inflammatory cytokines using a U-PLEX multiplexed cytokine array and a MESO QuickPlex SQ120 plate-reader.

### 2.6. In Vivo Study Outline

Permission to perform this study was granted by the ethical committee of the canton of Graubünden in Switzerland (approval number 05/2015), and the experiment was carried out in an AAALAC-accredited research institute. Our previously reported model of ODRI in the rat proximal tibia was used, utilizing custom PEEK screws [25,26]. A total of 36 rats were included in the study (an overview of grouping can be seen in Table 1). At 20–24 weeks of age, PEEK screws, either sterile or colonized with *S. epidermidis*, were implanted into the left tibia, as described below. In one group of animals, 2 mg of sterile titanium WPs were added to the implant site. Post-implantation, tibiae were monitored with in vivo microCT at seven time-points over a 28-day period.

### 2.7. Implant Design and Manufacturing

Custom-made screws (5 mm length, 1.5 mm diameter) were machined from medical grade PEEK containing 20% (*w*/*w*) barium sulphate (material supplied by Invibio Biomaterials Ltd., Thornton-Cleveleys, UK) by RISystem AG, Davos, Switzerland. Before use, screws were cleaned by ultrasonication (3 washes of 15 min each) in isopropanol, 70% ethanol, and ddH_2_0. All screws were steam sterilized for 20 min in an autoclave at 121 °C before implantation.

### 2.8. Bacterial Inoculum Preparation for In Vivo Study

The *S. epidermidis* 103.1 strain was cultured as described above. The bacterial inoculum was introduced to the rats on pre-contaminated screws prepared immediately prior to each surgery. On the day of surgery, *S. epidermidis* overnight cultures were centrifuged (2500× *g* for 10 min), washed in phosphate buffered saline (PBS) twice and then adjusted to an optical density of 0.50 (±0.01) at 600 nm. The threaded portion of the screw was submerged and incubated statically at room temperature for 25 min. Test screws were inoculated in parallel within each series of experiments. A quantitative assessment of *S. epidermidis* adhesion to the test screw was performed by sonication in PBS (3 min) followed by serial dilution, plating on 5% horse blood agar (Oxoid) and incubation overnight at 37 °C. The target inoculum for each screw was 1.5 × 10^6^ CFU/screw (range: 0.9–2 × 10^6^ CFU/screw). All screws were implanted within 30 min of preparation.

### 2.9. Animal Welfare, Observation and Euthanasia

Skeletally mature, adult female, specific pathogen-free (SPF) Wistar rats, purchased from Charles River (Germany), were used in this study, and were housed until skeletal maturity (20–24 weeks). Animal monitoring, care and potential exclusion criteria were as previously described [25,26,28]. Animals were randomly allocated to their group (Table 1). All animals were scanned by microCT immediately following surgery (day 0—to confirm appropriate positioning of screw) and at 6 further time-points post-surgery (see details below). At the conclusion of the study on day 28, animals were euthanized by intracardiac injection of pentobarbital under isoflurane anesthesia.

### 2.10. Anesthesia, Surgery and Medication Administration

Anesthesia and surgery were performed as previously described [25,26,28]. In brief, screw insertion surgery was performed at 20–24 weeks, using the previously described protocol [29]. Under isoflurane anesthesia and buprenorphine and carprofen analgesia, a sterile or a colonized screw (for the control and the infected groups, respectively) was inserted in the proximal tibia 2 mm distal to the growth plate. The experimental groups receiving titanium WPs had 10 µL of a 200 mg/mL titanium WP solution in PBS (corresponding to a total dose of 2 mg) injected into the pre-drilled hole prior to screw implantation. Further subgroups of animals infected with *S. epidermidis* were treated with a combination antibiotic regimen (25 mg/kg rifampicin plus 30 mg/kg cefazolin, *s.c.*) twice daily from day 7 after screw implantation, for a period of 14 days, followed by a 7-day washout period, to prevent false negative results in the quantitative bacteriological analysis.

### 2.11. In Vivo MicroCT and Image Processing

The evolution of bone structure was assessed using time-lapsed in vivo microCT (VivaCT 40, Scanco Medical AG, Bruettisellen, Switzerland) immediately post-operatively and at days 3, 6, 9, 14, 20 and 28, as previously described [25,28]. Briefly, animals were scanned under isoflurane anesthesia and a 10 mm long ROI, with a ø25.6 mm field of view and centered on the implanted screw was scanned at a nominal resolution of 25 µm. The X-ray tube was operated at 70 kVp voltage, 114 µA current, using 220 ms integration time and acquiring 1000 projections over 180° rotation [25]. Time-lapse microCT image processing was performed to compute bone fraction (BV/TV), bone formation (BF) and bone resorption (BR) rates in a region of interest within 700 µm from the screw surface. Periosteal reaction was quantified within the medial periosteal region 2 mm distal and proximal from the screw head. All image processing and analysis were performed with EasyIPL (easyipl.com, access date: 20.08.2019), a high-level library of macros using the scanner software (SCANCO Image Processing Language, IPL and OpenVMS Digital Command Language, DCL).

### 2.12. Bacteriology

Following euthanasia, the respective tibiae were dissected, and the screws and bones were collected in separate, sterile containers with sterile PBS, as previously described [26,30]. Samples were stored at 4 °C until processing. Bacteria numbers adhering to the *S. epidermidis*-contaminated screws were determined by sonicating the screws (3 min) before performing serial dilutions and viable bacteria counts on blood agar. The entire tibia from each animal was mechanically homogenized (Omni Tissue Homogenizer and Hard Tissue Homogenizing tips, Omni International, Kennesaw, GA, USA) and resident bacteria were quantified by serial dilution on blood agar. Soft tissue samples were processed in the same manner. All agar plates were incubated for 24 h at 37 °C, and all growth was checked for contamination or signs of co-infection. Animals were considered as infected when at least one sample (bone, soft tissue, or screw) was culture positive. Identification of *S. epidermidis* 103.1 in culture-positive samples was performed for at least one colony from each culture positive animal, using Random Amplification of Polymorphic DNA (RAPD) PCR [31] following comparison with the original *S. epidermidis* 103.1 strain.

### 2.13. Statistical Analysis

Data are reported as mean ± SD unless stated otherwise. Two-way ANOVA with Sidak’s multiple comparison test was used to compare the antibiotic activity in vitro between the different conditions, and one-way ANOVA with Dunn’s multiple comparison test was used to analyze quantitative CFU data within the group (with or without antibiotic). Two-way ANOVA with Tukey’s multiple comparison test was used to analyze CFU counts in co-cultures of neutrophils, *S. epidermidis* and WPs, while ordinary one-way ANOVA with Dunnett’s multiple comparison test was used to analyze changes in MPO release between groups. Analyses of the cytokine expression was performed by one-way ANOVA with Dunn’s multiple comparison test. One-way ANOVA with Dunn’s multiple comparison test was used to analyze quantitative CFU data in vivo. The Fisher exact test was used to check for differences in proportions of infected animals between groups. Two-way ANOVA with Tukey’s multiple comparison test was used to analyze changes in bone morphometric parameters over time. Threshold for statistical significance was set as *p* < 0.05. All calculations were performed using GraphPad Prism software (GraphPad Software, Inc., La Jolla, CA, USA).

## 3. Results

### 3.1. Antibiotic Challenge of Biofilm on Wear Particles

**Figure 1:**
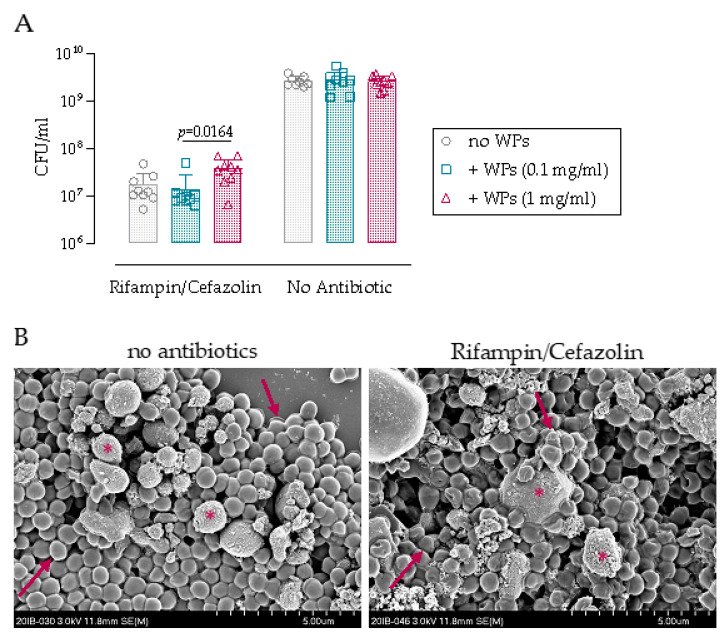
In vitro antibiotic killing activity is reduced when bacteria are cultured on titanium WPs. (**A**) Antibiotic killing activity of rifampin/cefazolin (both 100 µg/mL) against *S. epidermidis* cultured for 24 h with 0.1 or 1 mg/mL WPs. Data represent the CFU/mL with and without antibiotic treatment. Statistical analysis using 2-way ANOVA with Sidak’s multiple comparison test was performed between the rifampin/cefazolin and no antibiotic group (*p* < 0.0001, not shown) and one-way ANOVA with Dunn’s multiple comparison test within the rifampin/cefazolin family, *n* = 3. (**B**) SEM images of bacteria growing for 24 h in the presence of 1 mg/mL WPs and after 24 h in the absence (left) or presence of rifampin/cefazolin (right). Arrows indicate bacteria; asterisks indicate the WPs.

We first investigated whether the presence of titanium WPs influences *S. epidermidis* growth and the efficacy of rifampin/cefazolin combination in vitro. The growth of *S. epidermidis* reached a maximal density of 2.6 × 10^9^ CFU/mL for all the conditions (no WPs, or WPs at 0.1 or 1 mg/mL) indicating that WPs do not have any inherent antimicrobial activity (Figure 1A). Rifampin/cefazolin exposure resulted in an approximate 2-log CFU decrease in *S. epidermidis* under all conditions (no WPs, or WPs at 0.1 or 1 mg/mL) compared to the untreated control (Figure 1A, *p* < 0.0001). Antibiotic activity was similar for the “no WPs” and “0.1 mg/mL WPs” groups, while significantly less activity was detected in the presence of WPs at 1 mg/mL compared with 0.1 mg/mL (*p* = 0.0164). The bacteria grown for 24 h in the presence of 1 mg/mL of WPs were also imaged by SEM to assess morphological changes in response to antibiotic exposure (Figure 1B). Rifampin/cefazolin treatment induced minor morphological changes to the *S. epidermidis* grown on the WPs. Taken together, these data suggest that bacteria cultured in the presence of higher concentrations of WPs are more resistant to antibiotic therapy and appear more challenging to eradicate.

### 3.2. Impact of WPs on Neutrophil and PBMC Function

**Figure 2:**
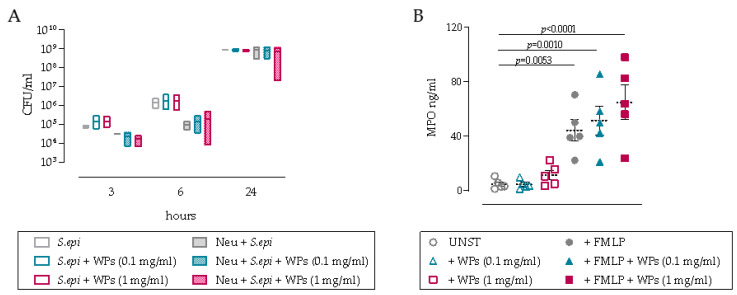
WPs decrease the inhibitory effects of neutrophils on *S. epidermidis* growth and induce MPO release. (**A**) Primary neutrophils killing activity expressed as CFU/mL calculated after 3, 6 or 24 h of incubation with *S. epidermidis* in the absence or presence of 0.1 or 1 mg/mL WPs. Statistical analysis by 2-way ANOVA with Tukey’s multiple comparison test (*n* = 3 independent donors); (**B**) myeloperoxidase (MPO) release from primary neutrophils after 24 h culture in the absence or presence of 0.1 or 1 mg/mL WPs, and after activation with 100 nM N-formyl-Mel-Leu-Phe (FMLP) (*n* = 5 independent donors). Statistical analysis by ordinary one-way ANOVA with Dunnett’s multiple comparison test, *n* = 5.

The impact of WPs on immune cell function was then assessed by measuring the anti-bacterial efficacy of neutrophils in co-cultures with *S. epidermidis* and also by assessing MPO release. Neutrophil bacterial killing activity peaks at 6 h in co-cultures with *S. epidermidis* with 1-log CFU reduction, with limited or no anti-bacterial effects observed at 3 or 24 h, respectively (Figure 2A). The addition of WPs, at any concentration, did not significantly affect the anti-bacterial efficacy of neutrophils, with high variability evident in these primary neutrophil co-cultures. Consistent with this lack of anti-bacterial efficacy, MPO release was not significantly affected by the presence of WPs, although there was a trend for increased MPO release in cultures with 1 mg/mL WPs compared to the unstimulated control (Figure 2B). Treatment with the chemotactic peptide FMLP to activate neutrophils markedly increased MPO release from primary neutrophils; however, no significant differences were observed in the presence of WPs (Figure 2B).

**Figure 3:**
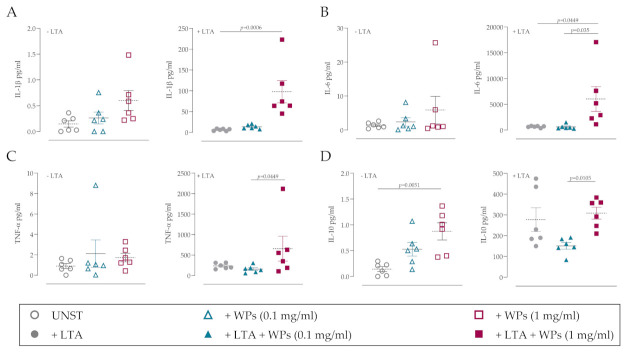
WPs induce pro-inflammatory cytokine release from PBMCs in the presence of bacterial-derived factors. Peripheral blood mononuclear cells (PBMCs) were incubated for 24 h with 0.1 or 1 mg/mL WPs in the absence or presence of 10 µg/mL lipoteichoic acid (LTA). Cytokine levels in conditioned media were assessed using multiplexed ELISA: (**A**) IL-1β; (**B**) IL-6; (**C**) TNF-α; (**D**) IL-10. Left panels are unstimulated cultures; right panels are following LTA treatment. Statistical analysis by one-way ANOVA with Dunn’s multiple comparison test; *n* = 3 independent donors performed in duplicate.

To further investigate the influence of WPs on primary immune cell populations, PBMCs were incubated with WPs and/or LTA for 24 h. The pro- and anti-inflammatory cytokine responses were assessed using a multiplexed cytokine array. The presence of WPs alone did not significantly induce the production of the pro-inflammatory cytokines IL-1β, IL-6 and TNF-α compared to the unstimulated control (Figure 3A–C; left panels), although the anti-inflammatory cytokine IL-10 was significantly increased (Figure 3D; left panel). However, when WPs were present in combination with LTA, WPs significantly increased the release of IL-1β, IL-6 and TNF-α (Figure 3A–C; right panels). Taken together, this suggests that the presence of WPs may influence immune cell function, particularly in the presence of bacterial-derived factors.

### 3.3. In Vivo Bacteriology

**Figure 4:**
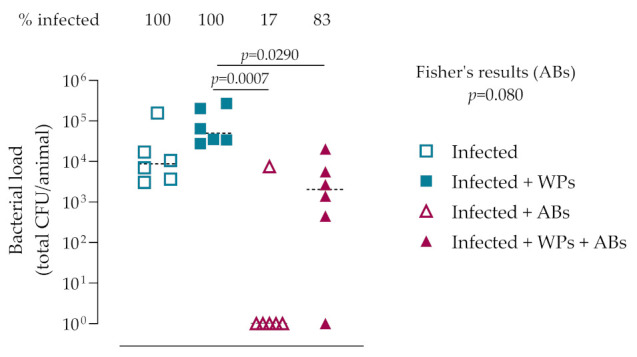
Wear particles increase bacterial burden and diminish antibiotic efficacy in vivo. A sterile or *S. epidermidis*-inoculated screw was implanted into the proximal tibia of female Wistar rats in the presence of absence of 2 mg titanium WPs. In the antibiotic-treated groups (Infected + ABs), animals were administered rifampin plus cefazolin. At euthanasia (day 28) the number of bacteria (CFU) was determined by quantitative bacteriology. Results shown are from individual animals (*n* = 6 per group). The mean is indicated by the horizontal bar. Culture-negative samples were arbitrarily assigned a value of 1 for the purposes of displaying on a log_10_ axis. Statistical analysis performed using the Kruskal–Wallis test with Dunn’s multiple comparison test. A Fisher’s exact test (indicated) was performed to compare proportions of infected animals between antibiotic-treated groups in the absence or presence of WPs.

We then assessed the effect of WPs influencing the severity of a *S. epidermidis* implant-related infection in vivo and the response of the infection to antibiotic treatment. Quantitative bacteriology revealed that all animals receiving *S. epidermidis*-inoculated screws remained infected after 28 days. The presence of titanium WPs at the implant site resulted in a trend for an approximate three-fold increase in bacterial burden compared to control infected animals, although this was not statistically significant (mean CFU: Infected = 3.3 × 10^4^ ± 6.09 × 10^4^ CFU vs Infected + WPs = 1.05 × 10^5^ ± 1.04 × 10^5^; *p* = 0.708; Figure 4). This suggested that the WPs might influence host immune responses and/or provide an increased surface area for the bacteria to colonize. A 2-week period of combination antibiotic therapy (rifampin plus cefazolin; days 7–21) successfully cleared the infection in the majority (5/6) of control animals, thus demonstrating the efficacy of this treatment regimen in our model system. The lack of bacterial clearance in the one remaining infected animal was not due to the emergence of rifampin resistance in this animal, since zone of inhibition assays confirmed that the recovered bacteria were equally susceptible to rifampin as the parental strain (data not shown). Although the antibiotic therapy significantly decreased CFU counts in WP-treated animals (*p* = 0.029), five out of animals remained infected at euthanasia, thereby demonstrating that the antibiotic treatment was markedly less effective at clearing the infection when WPs were present in the vicinity of the implanted screw.

### 3.4. MicroCT

**Figure 5:**
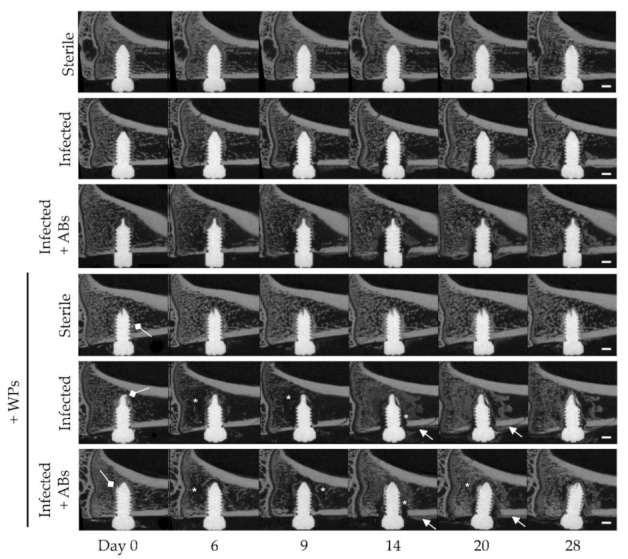
MicroCT revealed increased *S. epidermidis*-induced osteolysis in the presence of WPs. A sterile or *S. epidermidis*-colonized screw was inserted in the unicortical hole created in the proximal tibia of female Wistar rats. Further subgroups included animals receiving 2 mg of sterilized titanium WPs into the screw hole and, in animals receiving the *S. epidermidis*-colonized screws, a combination antibiotic regimen (+ABs) was administered on days 7–21, followed by a 7-day washout period. The evolution of bone structure was assessed by in vivo microCT imaging at day 0, 6, 9, 14, 20 and 28. *n* = 6 per experimental group. Asterisks highlight osteolysis around the screw, arrows represent thickening of periosteal regions in proximity of the screw, and white arrows (diamond-headed arrow) represent WPs. Scale bar = 1 mm.

The impact of WPs on *S. epidermidis*-induced osteolysis, periosteal reaction volume, and bone resorption/formation rates was determined by longitudinal in vivo microCT over 28 days. *S. epidermidis* induced osteolysis around the screw, observed within 6 days and peaking at 9 days post-implantation (Figure 5, asterisks). By day 14, a marked thickening of the periosteal region in the vicinity of the screw was also observed, followed by extensive deposition of mineralizing repair tissue between days 20 and 28 (Figure 5, arrows). Although antibiotic activity was shown to successfully treat the infection, osteolysis and reparative bone formation were not improved in the antibiotic-treated group. Although the presence of WPs in the implant site did not affect the bone responses in the non-infected animals, greater osteolytic responses were observed in infected rats receiving WPs (Figure 5, asterisks). Antibiotic treatment did not alter the osteolytic or reparative responses to infection in WP exposed rats. Taken together, this suggests that the presence of WPs exacerbates infection-induced bone destruction.

**Figure 6:**
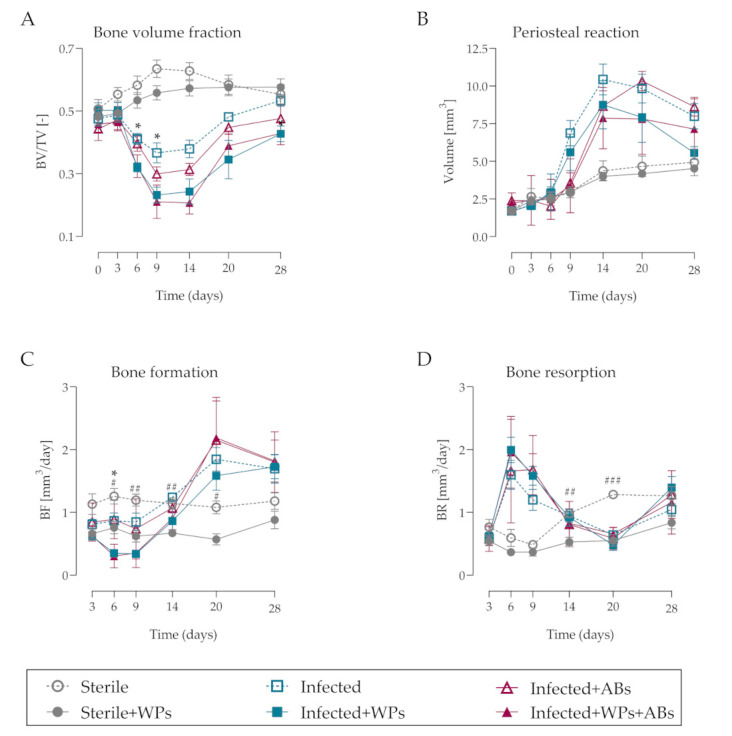
Quantitative morphometric analyses reveal changes in bone fraction, periosteal reaction bone formation and bone resorption in response to *S. epidermidis* infection and presence of wear particles over 28 days. *S. epidermidis*-inoculated screws induce marked changes in (**A**) bone fraction (BV/TV), (**B**) periosteal reaction, (**C**) bone formation (BF) and (**D**) bone resorption (BR) over time, which is affected by the presence of titanium WPs. Data shown are the mean ± SEM. Statistical analysis was by 2-way ANOVA with Tukey’s multiple comparison test: * indicates a significant difference between Infected and Infected+WPs (*p* < 0.05); # indicates a significant difference between Sterile and Sterile + WPs (# *p* < 0.05; ## *p* < 0.01; ### *p* < 0.001).

Quantitative morphometric analysis of the microCT data revealed dynamic changes in bone tissue over the course of the infection. Osteolysis around the screw, shown as a reduction in bone volume/total bone volume (BV/TV) was evident in the animals receiving a *S. epidermidis*-inoculated screw as early as day 6 and achieved peak osteolysis at day 9 (Figure 6A). *S. epidermidis* induced an approximate 23% reduction in BV/TV values in the vicinity of the screw at day 9, while the presence of WPs, BV/TV profoundly reduced by approximately 54% at the same time-point (Figure 6A). Although BV/TV values in the infected animals recovered to those of non-inoculated animals at day 28, the presence of WPs also impaired reparative responses in the inoculated animals, with a significant decrease in BV/TV remaining at day 28 (*p* = 0.033). Interestingly, the presence of WPs in non-infected animals resulted in no significant changes in BV/TV. Furthermore, antibiotic treatment did not affect BV/TV values in infected animals, either in the absence or the presence of WPs.

The pronounced effects of WPs in the infected animals for enhancing osteolytic responses were not associated with any significant changes in the degree of periosteal reaction volume throughout the 28-day experimental period (Figure 6B), although there was a trend for diminished periosteal reaction volume in the infected animals with WPs at day 9 (*p* = 0.052).

Analysis of bone formation and resorption rates indicated that the presence of WPs in the infected animals significantly decreased bone formation at day 6, with an overall trend for decreased bone formation up to day 14 (Figure 6C). Similar trends were evident in the infected animals receiving antibiotic treatment (Figure 6C). Interestingly, bone formation rates were also significantly decreased between days 6 and 20 in the sterile animals receiving WPs. Although infection induced marked increases in bone resorption at days 6 and 9, no differences were observed between infected animals in the absence or presence of WPs (Figure 6D). Similarly, no differences in bone resorption were observed in the infected animals receiving antibiotic therapy in the absence or presence of WPs (Figure 6D). Concerning the non-inoculated animals, wear particles also significantly decreased bone resorption at days 14 and 20, in addition to their negative effects on bone formation. Taken together, this suggests that WPs predominantly exacerbate infection-induced osteolysis by inhibiting bone formation rather than increasing osteolytic responses.

## 4. Discussion

TJA surgeries are a mainstay of current orthopedic practice, but the long-term survival of implants may be compromised due to the generation of WPs, which can drive inflammatory responses and contribute to aseptic loosening. However, the consequences of WPs for influencing the course of a low virulence bacterial infection, or the response of such an infection to antibiotic therapy, remains poorly understood. In this study, we determined that WPs enhance pro-inflammatory cytokine production by immune cells in vitro and exacerbate infection-induced osteolysis and dramatically diminish antibiotic efficacy in vivo.

Perhaps of greatest concern from our study is our finding that WPs negatively impact antibiotic efficacy in vivo, as shown from the bacteriological analyses of the retrieved tissues. While the specific mechanism underlying such a negative interaction between WPs and antibiotics is currently unknown, one potential explanation is that the presence of WPs interferes with host immune cell function, which is necessary for the effective eradication of viable bacteria present at the infection site. It has previously been shown that ultra-high molecular weight polyethylene (UHMWPE) WPs can inhibit the antibacterial activity of neutrophils in vitro, whereby internalization of WPs results in an inability of neutrophils to further phagocytose bacteria such as *S. aureus* [32,33]. Furthermore, cobalt-chromium and UHMWPE WPs have also been demonstrated to contribute to bacterial persistence in mice using a subcutaneous air-pouch model [34], although the efficacy of antibiotic therapy was not assessed in this system. Thus, it appears that, in the context of our specific preclinical model, that effective clearance of a *S. epidermidis* implant-related infection requires both antibiotic treatment and non-compromised immune cell function.

It is also apparent from our study that the presence of endotoxin-free titanium WPs at the implant site per se does not contribute to osteolytic responses in the absence of additional stimuli, such as bacterial-derived products such as LTA. Indeed, sterile implanted screws in the presence of WPs displayed minimal changes to bone parameters as assessed by longitudinal microCT scanning. This is in contrast with numerous in vivo studies demonstrating osteolytic responses to WPs in the absence of bacterial infection [35,36,37], although this likely reflects the prevalence and specific experimental context of the murine calvarial osteolytic model that is widely used for assessing WP-induced osteolysis. The contribution of bacterial-derived endotoxins for driving osteolytic responses, either due to the use of contaminated WPs, or via binding from systemic sources following WP implantation [38], appears to be a major stimulus for WP-induced osteolysis [39,40,41]. However, our study and a variety of others support the notion that WPs exacerbate pro-inflammatory responses induced by bacterial-derived factors (pathogen-associated molecular patterns; PAMPs). Biofilm components and WPs induce neutrophil recruitment in vivo by promoting local IL-8 release (a potent neutrophil chemoattractant) [42] via a mechanism which also appears to involve IL-1β production by macrophages and IL-1 receptor signalling [43]. Consistent with our study, elevated levels of pro-inflammatory cytokines and chemokines induced by prosthetic WPs have been shown in several in vitro and in vivo studies [44,45], and also in tissue retrieved during revision arthroplasty in patients with aseptic loosening when compared to patients with implant failure due to mechanical causes [45]. Thus, WPs appear to markedly contribute to pro-inflammatory responses, capable of driving excessive osteolysis particularly when PAMPs are also present at the implant site.

Osteolytic responses to WPs, in the presence or absence of PAMPs, appear to be driven via Toll-like receptor (TLR) signaling, predominantly TLR-2 and TLR-4 [46,47]. Indeed, using mice deficient in the TLR signaling adapter protein MyD88 (myeloid differentiation factor 88), Pearl et al. revealed that WP-induced osteolytic responses were diminished in MyD88-deficient mice compared to wild-type mice. Furthermore, WPs induced decreased TNF-α production in MyD88-deficient macrophages, with macrophage-derived TNF-α and NFκB signaling previously identified as crucial mediators of WP-induced pro-inflammatory responses and osteolysis in vivo [35].

The identification of crucial signaling pathways involved in WP-induced pro-inflammatory responses and osteolysis therefore raises the possibility that pharmacological modulation of these pathways could prevent detrimental responses to WPs in patients. A variety of drugs have displayed efficacy in preventing osteolytic responses to WPs in vivo, particularly anti-resorptive agents targeting osteoclasts, such as bisphosphonates and osteoprotegerin (OPG) [36,37], and agents targeting pro-inflammatory signaling such as IL-1β [43] or NFκB activation [48], although these were assessed in the absence of a bacterial infection. Indeed, we have previously assessed the efficacy of the bisphosphonate zoledronic acid [26] and the non-steroid anti-inflammatory drug carprofen [30] for affecting osteolytic responses to *S. epidermidis* infection, but found both drugs to diminish antibiotic efficacy. However, given the increasing realization that a majority of routinely removed implants are contaminated with bacterial DNA (and thereby may be incorrectly classified as aseptic) [49,50], prosthetic joint loosening may be prevented by effective antibiotic therapy. In support of this, the antibiotics enoxacin [51] and rifampin [52] have both been demonstrated to prevent WP-induced osteolysis in vivo, suggesting that hematogenous seeding of bacteria or PAMPs on WPs may be prevented by antibiotic therapy, and thus may be an effective means for preventing prosthetic joint loosening in patients. However, the failure of antibiotic therapy (including rifampin) in our preclinical model may reflect the specific timing of antibiotic administration (7 days post-screw implantation). It remains to be determined whether the efficacy of our antibiotic regimen for preventing the WP-induced exacerbation of osteolysis could be improved by earlier administration.

Regarding the limitations of our study, it has previously been reported that microCT-based scanning was incompatible with titanium WPs for the assessment of osteolysis in the murine calvarial model [36]. Although titanium WPs were evidently present in our microCT-based assessment, this did not prevent the assessment of osteolytic changes in our model and may even underestimate the extent of the WP-induced osteolysis we observed, due to the potential classification of WP as mineralized tissue or partial volume effects. In addition, we only assessed one material (titanium) for assessing the impact of WP on bone changes and the response to antibiotic therapy. Given the known influence of material surface chemistry and size on the interactions with bacteria or bacterial-derived factors, the choice of WP material may indeed influence our observation regarding antibiotic efficacy. Furthermore, we only assessed one dosing regimen (initiated on day 7 for 2 weeks) of a combination antibiotic therapy including rifampin, based on the efficacy of rifampin for affecting biofilm-resident Staphylococci [53]. As a clinically relevant antibiotic therapy, we feel this is an appropriate choice for assessing antibiotic efficacy, although it would be interesting to assess whether WPs also negatively influence antibiotic efficacy when administered in a peri-operative prophylactic context.

## 5. Conclusions

In conclusion, we demonstrate the temporal changes that WPs induce in the osteolytic response to a low virulence *S. epidermidis* infection in vivo and highlight the diminished antibiotic efficacy when WPs are present in the vicinity of the implant. Therefore, strategies aimed at minimizing the generation of WPs and/or the colonization of the WPs with low-virulence bacteria or PAMPs should be considered to minimize the risk of osteolysis occurring following the implantation of prosthetic joints.

## Figures and Tables

**Table 1:** Overview of study design.

Group		Inoculum	Group Size
1	Control	Sterile	6
		*S. epidermidis*	6
		*S. epidermidis* + antibiotics	6
2	Wear particles	Sterile	6
		*S. epidermidis*	6
		*S. epidermidis* + antibiotics	6
		TOTAL	36

## Data Availability

Not applicable.
